# Young and old adult brains experience opposite effects of acute sleep restriction on the functional connectivity network

**DOI:** 10.1162/IMAG.a.1278

**Published:** 2026-06-18

**Authors:** Josh Neudorf, Leanne Rokos, Kelly Shen, Brianne Kent, Anthony R. McIntosh

**Affiliations:** Institute for Neuroscience and Neurotechnology, Simon Fraser University, Burnaby, Canada; Centre for Social Sciences, Athabasca University, Athabasca, Canada; Department of Biomedical Physiology and Kinesiology, Simon Fraser University, Burnaby, Canada; Rotman Research Institute, Baycrest Health Sciences, Toronto, Canada; Department of Psychology, Simon Fraser University, Burnaby, Canada

**Keywords:** resting-state functional magnetic resonance imaging, dynamic functional connectivity, graph theory, healthy ageing, acute sleep restriction

## Abstract

Chronic, long-term sleep loss is detrimental to brain health and cognitive ability. However, older adults are affected differently by acute, short-term loss of sleep than young and middle-aged adults. Older adults are more resilient to the effects of acute sleep loss and, depending on the cognitive domain, may be completely unaffected while younger adults suffer. To elucidate the brain network responses to sleep loss underlying these cognitive differences between age groups, we investigated the static and dynamic functional connectivity effects of acute sleep restriction (sleep limited to 3 hours) and how these effects differ between younger adults (20–30 years) and older adults (65–75 years). We found a functional connectivity subnetwork that was primarily strengthened in younger adults after acute sleep restriction but weakened in older adults after acute sleep restriction. Similar crossover interactions were consistently observed in further analyses of functional connectivity degree, modularity, and dynamic functional connectivity state fractional occupancy. Our findings demonstrate that the effect of acute sleep restriction on older adults is fundamentally different from that on younger adults. These results most strongly support the compensation theory of ageing, which predicts a fundamental shift in the effects of acute sleep loss, rather than a mere dampening of the sleep benefits experienced by younger adults.

## 1 Introduction

In fast-paced, work-centric cultures, it can sometimes feel as though there is not enough time in the day. We may resent our body’s daily need for sleep. In many cases, the combined demands of life, including work and family, lead to sacrificing sleep for the sake of finding more time. However, this loss of sleep is indeed a sacrifice, which can come at a cost. Beyond the well-known short-term effects of irritability and diminished cognitive ability, cumulative loss of sleep over the lifespan takes its toll, negatively impacting the brain and body. Poor sleep has the potential to increase the risk of cardiovascular disease, hypertension, diabetes, weight problems, colorectal cancer, and all-cause mortality, to name a few of the possible repercussions (see Medic et al., 2017 for a review). Poor sleep also adds up over time to negatively impact the brain. Middle-aged adults with short nights of sleep have more β-amyloid in their brains (Spira et al., 2013), which in many cases precedes cognitive decline and Alzheimer’s disease (Bateman et al., 2012). Indeed, many longitudinal studies have identified poor sleep in middle age as a predictor of cognitive decline and Alzheimer’s disease later in life (Ferrie et al., 2011; Kulmala et al., 2013; Lobo et al., 2008; Stenfors et al., 2013; Virta et al., 2013).

The acute, short-term effects of sleep loss on cognitive performance and the brain are more nuanced than these chronic, long-term effects. Young and middle-aged adults’ attention, executive control, working memory, episodic memory, and mood are impacted negatively when sleep is missed (acute sleep restriction, where sleep is limited, and acute sleep deprivation, where no sleep is attained; e.g., Boland et al., 2017; Krieg et al., 2001; Kronholm, 2012; Pilcher & Huffcutt, 1996; Regestein et al., 2004; Scullin & Bliwise, 2015; Sternberg et al., 2013). However, there are also temporary positive effects of skipping or limiting a night of sleep. For example, across multiple studies, the antidepressant effects of acute sleep deprivation have been well replicated. Around half of young and older adults with depression symptoms respond positively to acute sleep deprivation, noting a reduction in their symptoms (see Thase et al., 2017 for a review). Furthermore, there are many effects of acute sleep deprivation that have been observed in younger adults but have not been replicated in older adults or are markedly weaker. Across multiple cognitive domains and many studies, it has been demonstrated that older adults are resilient to the cognitive effects of acute sleep deprivation (Gerhardsson et al., 2019; Scullin & Bliwise, 2015; Sutter et al., 2012). Older adults are also more resilient to the negative effects of acute sleep deprivation on mood (healthy adults generally respond with more negative mood in response to sleep deprivation, as opposed to the exception noted above for some adults with depression symptoms; Schwarz et al., 2019).

There have been a number of theories developed to explain why older adults are more resilient to acute sleep loss (restriction and deprivation) than younger adults (see Scullin & Bliwise, 2015 for a review). The most prominent of these theories include the reduced sleep need, functional weakening, and compensation theories. The reduced sleep need theory suggests that older adults need less sleep than they did as younger adults. Whereas earlier in the lifespan, sleep was an important process for maintaining cognitive ability, as the amount of learning decreases with age, so too does the need for sleep to consolidate that learning (Cirelli, 2012; Feinberg, 2000). A second theory, the functional weakening theory, suggests that the ageing brain undergoes multiple negative changes that undermine the ability of sleep to perform the restorative, constructive role it once held (Edinger et al., 2000; Kronholm, 2012; McCrae et al., 2012; Pace-Schott & Spencer, 2011; Schmidt et al., 2012; Scullin, 2013; Spiegel et al., 1986). For this reason, not only do older adults not need additional sleep to maintain their baseline cognitive performance, but furthermore, an increase in sleep will not produce benefits because the brain architecture that once supported the sleep–cognition relationship has deteriorated. Finally, the compensation theory suggests the brain undergoes fundamental adaptations with age to compensate for the changing roles of particular sleep stages for cognition, which affect resilience to acute sleep loss (Nemeth et al., 2013; Park & Reuter-Lorenz, 2009; Peters et al., 2008; Salas et al., 2014; Spencer & Pace-Schott, 2013). This theory predicts that the effects of sleep loss will be fundamentally different for older adults, rather than simply being a less pronounced version of what is observed for younger adults. Going beyond the cognitive effects of sleep loss across the lifespan to investigate the brain changes that underlie these differences may provide additional insight into which of these theories are most consistent with the evidence.

A large body of research has found that acute sleep loss impacts young adult brain regions and networks in many ways (e.g., Ben Simon et al., 2017; Caldwell et al., 2004; Chee & Zhou, 2019; Chengyang et al., 2017; Dai et al., 2020; Drummond et al., 2000; Huang et al., 2022; Tian et al., 2024; Xu et al., 2018; Yeo et al., 2015), but little is known about how the effects of acute sleep loss present in the brain networks of older adults. This research has primarily been performed on young adult brains, and comparing these effects across age groups remains a gap in the literature. One study comparing the effects across age groups focused on the interaction between acute sleep restriction (sleep limited to 3 hours) and age on the functional connectivity between a small number of large functional networks but did not identify a significant interaction (Nilsonne et al., 2017). However, there are more advanced methods that could be used to investigate the brain as a finer-scaled network with hundreds of regions to identify network changes at the scale of the regions and connections between regions and at the whole-brain scale utilizing a modularity analysis. Furthermore, dynamic functional connectivity changes have not been investigated in this context, which would provide further insights.

Functional connectivity analyses investigate the coactivation of regional brain activation, using, for example, functional magnetic resonance imaging (fMRI) blood oxygen level-dependent (BOLD) signal, to calculate a measure of functional correlation between brain regions. Graph theory is the field of mathematics focused on networks and has been applied to brain network data with tremendous success (Sporns, 2018). Graph-based analysis methods have been developed expressly for the comparison of population groups’ brain network data, including the network-based statistic for comparing parcellated brain connectivity matrices (Zalesky et al., 2010) and multivariate distance matrix regression for comparing voxel-wise brain connectivity matrices (Shehzad et al., 2014). In addition to studying static functional connectivity as described above, the dynamics of functional connectivity should also be investigated, as it has been demonstrated that functional connectivity patterns rapidly change over time, even at rest, and that these dynamics are meaningful as they are associated with cognitive ability in young and older adults (Cabral et al., 2017; Cohen, 2018; Hutchison et al., 2013; Neudorf et al., 2025) and disease (Cohen, 2018; Dautricourt et al., 2022; Jin et al., 2023; Zang et al., 2024). We propose to use these methods to investigate how the functional brain network effects of acute sleep restriction differ between younger and older adults. We hypothesize that there should be a significant interaction between the effects of acute sleep restriction and age, considering the consistent findings that older adults are less affected cognitively by acute sleep deprivation and restriction than younger adults. The theories that have been developed to explain this phenomenon produce different hypotheses for the nature of this interaction. The reduced sleep need and functional weakening theories propose reduced beneficial effects of sleep and would predict a reduced brain network effect of acute sleep restriction in older adults. However, the compensation theory proposes that older adults undergo a fundamental change in how the brain relies on the various sleep stages in order to support cognitive abilities, such as memory consolidation. Under the assumptions of this theory, we expect to see more of a fundamental shift in the effect of acute sleep restriction on the brain network rather than simply a dampening of the effect seen in younger adults. A shift of this kind would not be surprising, given the fundamental differences from the young adult brain that we have observed in the functional communication and network organization of the ageing brain (Heisz et al., 2015; Neudorf et al., 2024, 2025).

## 2 Methods

### 2.1 Participants

Details about recruitment, ethics, and the full dataset can be obtained from the original dataset paper and a follow-up analysis paper (Nilsonne et al., 2016, 2017). Data were downloaded from OpenfMRI (https://openfmri.org/dataset/ds000201/). Participants were recruited in two different age groups that were either between the ages of 20 and 30 years (young adults; YA) or between the ages of 65 and 75 years (old adults; OA). The participants engaged in two separate scanning sessions on separate days, in which they either had a full night’s sleep or sleep restricted to 3 hours. The MRI procedure took place in the evening following the night of normal or restricted sleep (between 6 PM and 9 PM). In both conditions, ambulatory polysomnography was used to monitor sleep. In the restricted sleep condition, participants were asked not to sleep until 3 hours before they would normally wake up, and then wake up when they normally would. OA and YA had comparable regular sleep patterns, with YA having a mean of 8.389 hours of weekday sleep and OA having a mean of 8.305 hours of weekday sleep, *t*(40) = .286, *p* = .776, and YA having a mean of 8.617 hours of weekend sleep and OA having a mean of 8.655 hours of weekend sleep, *t*(40) = -.128, *p* = .899. The order of these sessions was counterbalanced, and took place approximately 1 month apart. Among other screening criteria, these participants had no past psychiatric or neurological illness and did not exhibit insomnia symptoms or excessive snoring.

### 2.2 MRI acquisition

Full details about MRI acquisition can be obtained from the original paper (Nilsonne et al., 2017). A General Electric Discovery 3T MRI was used. For the resting-state fMRI (rs-fMRI), echo-planar images were acquired with a flip angle of 75 degrees, TE of 30 ms, TR of 2.5 seconds, field of view of 28.8 cm, slice thickness of 3 mm, with a voxel size of 2.25 × 2.25 × 3 mm, acquiring 49 slices that were interleaved from the bottom up. In our analysis, from each participant, 173 volumes (totaling 7 minutes and 12.5 seconds) were used in order to equate the total number of volumes. The T1-weighted structural images utilized a sagittal BRAVO sequence with field of view of 24 cm and slice thickness of 1 mm acquired from the bottom up.

### 2.3 Preprocessing

Preprocessing of the MRI data utilized TheVirtualBrain-UK Biobank pipeline (Frazier-Logue et al., 2022), based on the UK Biobank pipeline (Littlejohns et al., 2020), and using the FMRIB Software Library (FSL; Jenkinson et al., 2012). TheVirtualBrain-UK Biobank pipeline accounts for issues related to atrophy in ageing brains using quality control methods outlined by Lutkenhoff et al. (2014). In addition, the pipeline produces parcellation-based time-series data from the fMRI volumes that were used in the functional connectivity analyses. The reader is referred to the preprocessing pipeline publication for the details about the preprocessing steps and order (Frazier-Logue et al., 2022). The data were parcellated using a combined atlas of the Schaefer 200 region atlas (Schaefer et al., 2018) and the subcortical Tian atlas (Tian et al., 2020). The subcortical regions included regions from the Tian Scale 1 atlas but with the hippocampus represented by the Tian Scale 3 atlas definition, with the two head divisions combined into a single parcel. This decision was made to include more granularity because of the known functional organization of the hippocampus along its anterior-posterior (A-P) Axis. We used the Scale 3 divisions into head, body, and tail (i.e., anterior to posterior divisions), but combined the head’s medial and lateral divisions to keep the divisions along the A-P Axis simple. A custom-trained *FSL FIX* classifier was provided to the pipeline, trained on 10 scans from the first scanning session, and 10 scans from the second scanning session, balancing the number of YA and OA participants and male and female participants, with *FIX* set to “aggressive” artefact removal (Griffanti et al., 2014). An alternative version of the pipeline was tried without the aggressive noise cleanup enabled, but many more subjects were excluded due to data quality issues using this option. For this reason, we opted to use the aggressive noise cleanup that produced more acceptable results.

The neuroimaging outputs were also subjected to manual quality control, which included manual inspection of brain extraction, segmentation, registration, masks, fMRI time-series plots, and FC matrices. Participants with missing data from either of the sleep conditions were excluded from the analyses. Structural T1 images were checked for motion artifacts and poor brain extraction, registration was checked for proper alignment, fMRI time series were inspected for residual motion artifacts, and FC matrices were inspected for poor separation of the values into intra- and inter-hemispheric quadrants and prominent “banding”, whereby a single region exhibits fairly constant values indicating motion artifacts and/or poor registration. Following the previous publication with these data, participants passing the previous quality control steps were checked to ensure that no more than 25% of volumes had frame-wise displacement greater than 0.5 mm. There were 48 YA and 36 OA in the sample, and following our quality control of the magnetic resonance imaging (MRI) data, we excluded 25 YA (13 female, 12 male) and 17 OA (9 female, 8 male), and included 23 YA (11 female, 12 male) and 19 OA (9 female, 10 male). The exclusions were not more heavily represented by either sex.

Time-series data from the pipeline were then bandpass filtered to remove frequencies outside of the range between 0.01 Hz and 0.1 Hz. To calculate the FC matrices, the time series were Z-scored across time separately for each region, the Pearson’s R correlation was calculated between each pair of regions, and then a Fisher Z-transformation was applied. Node degree was calculated as the sum of connection weights to each brain region, resulting in a single value for each brain region.

### 2.4 Dynamic functional connectivity

The rs-fMRI data were analyzed using a dynamic FC analysis approach called Leading Eigenvector Dynamics Analysis (LEiDA; Cabral et al., 2017). This method computes an FC matrix at each timepoint of the rs-fMRI scan using phase coherence connectivity (e.g., Deco et al., 2017; Deco & Kringelbach, 2016; Glerean et al., 2012; Ponce-Alvarez et al., 2015). In contrast to other dynamic FC methods that compare the full FC matrices across timepoints, LEiDA first computes the leading eigenvector of each FC matrix, making the method less susceptible to noise and better able to detect the recurrence of a particular state. The dynamic FC matrices are separated into distinct states by applying a clustering analysis on the leading eigenvectors for all subjects and timepoints, resulting in states that are common to all subjects. The ideal number of states was chosen based on an evaluation of the clustering analysis that maximized the Dunn’s score (Dunn, 1973), average Silhouette coefficient (Rousseeuw, 1987), and Calinski–Harabasz index (Caliński & Harabasz, 1974). With these states defined, each timepoint was then labeled according to which state the participant’s brain network was in at that timepoint, which allowed for calculation of the fractional occupancy of each state (probability of that state occurring at any given time), state dwell time (time spent in each state), and the transition probability matrices (probability of the brain state changing from a specific state to another, or maintaining the same state, represented as a K×K matrix where K is the total number of states). In summary, the LEiDA method identifies which state the brain is in at each timepoint, and allows for quantitative measures about how much time is spent in each state and how likely transitions are between these states.

### 2.5 Modularity

Modularity indicates the degree to which a network can be separated into distinct modules (i.e., communities). High modularity indicates that the network has strong connectivity within modules but weaker connectivity between modules. This measure was calculated using the Brain Connectivity Toolbox (BCT; Rubinov & Sporns, 2010) function *community_louvain*, with asymmetric treatment of negative weights from the FC matrices and resolution parameter set to 1 (variations of the resolution parameter were also tested at 0.75 and 1.25 to test robustness to this parameter). To test that the detected communities were more modular than would be expected by chance, 1000 null networks were produced using the *null_model_und_sign* function, which preserves weight and degree distributions as well as the approximated strength distributions. All networks’ modularity values surpassed every permuted null value with the resolution parameter set to 1 and 1.25, and 95.2% of networks surpassed permuted null values with the resolution parameter set to 0.75. In summary, the modularity method identifies an optimal modular organization of the functional connectivity network and quantifies the extent to which this network has strong connectivity within and weak connectivity between these modules.

### 2.6 Partial least squares (PLS)

Multivariate partial least squares (PLS) analysis (McIntosh & Lobaugh, 2004) was used to identify latent variables (LVs), each containing weights that describe the relationship of brain measures (e.g., FC, FC degree, fractional occupancy, dwell time, or transition probability) with age (between-subjects) and sleep condition (within-subjects). Mean-centred PLS analyses were performed by categorizing scans into four groups based on their age (YA or OA) and sleep condition (Restricted or Normal). PLS uses singular value decomposition to project the data matrix onto orthogonal LVs (similar to canonical correlation analysis). The significance of the identified LVs was determined via permutation testing. We report only the most reliable PLS weights as determined by bootstrap resampling, which is used to calculate bootstrap ratios (BSR), the ratios of the PLS weights (saliences) to their standard errors as determined by bootstrap resampling (Kovacevic et al., 2013; McIntosh & Lobaugh, 2004). The resampling procedures were performed using 1000 iterations for each. For the mean-centred PLS, brain scores are derived for each LV using the dot-product of the PLS weights for the brain metric and the values of the metric for each participant. The brain scores are similar to factor scores and indicate the degree to which a subject or group shows the pattern captured in the LV. In the present paper, we use these to convey the relative difference in LV expression between groups, using the mean brain score and the bootstrap estimated standard error. In summary, the PLS analysis identifies LVs in the brain data (FC, degree, and LEiDA metrics) associated with age and sleep.

To ensure that the PLS results were not affected by motion, additional models including motion as the independent variable were conducted. If these identified a significant LV, a follow-up analysis was performed computing the cosine similarity between the salience matrices of the motion model and the model of interest. If this cosine similarity was not significant, this indicated that the model of interest was not related to motion effects.

### 2.7 Network-based statistic

A network-based statistic (Zalesky et al., 2010) approach was used to identify an age group-related subnetwork from the functional connectivity sleep difference network (values from normal sleep subtracted from those obtained during restricted sleep). Each connection in this sleep difference network was used to compute an independent samples t-test between the two groups, which was thresholded at *p* < .01 (*t* > 2.7). A significant subnetwork was then checked for based on a permutation test with 5000 permutations comparing the empirical subnetwork size (number of involved regions) to those observed with permuted group labels. To test robustness to threshold variation, supplementary analyses were conducted with *t* > 2.5 and *t* > 2.9. In summary, the network-based statistic identifies part of the sleep restriction-related functional connectivity network (normal sleep network subtracted from the restricted sleep network) that differs between younger and older adults.

### 2.8 Multivariate distance matrix regression

Multivariate distance matrix regression (Shehzad et al., 2014) was used to identify voxel-wise differences in the FC connectivity profiles between age groups and sleep conditions. Voxels were resampled to 4 mm^3^ in MNI standard space and grey matter voxel-wise FC matrices were computed in the same manner used for the parcellation-based FC matrices. Group comparisons between YA and OA were tested on the voxel-wise FC sleep difference network, and sleep condition comparisons were tested on the voxel-wise FC network for YA and OA separately, using 15,000 pseudo-F-statistic permutations and 500 cluster size permutations (additional analyses were conducted with 750 and 1000 cluster size permutations to test robustness to this parameter). Surface visualizations of these results were produced using the *neuromaps* (Markello et al., 2022) implementation of volume-to-surface registration as proposed by Buckner et al. (2011) and implemented by Wu et al. (2018). In summary, the multivariate distance matrix regression highlights voxels that connect to the rest of the brain differently between groups (age groups) and/or conditions (e.g., sleep conditions).

## 3 Results

### 3.1 Functional connectivity

**Fig. 1.**
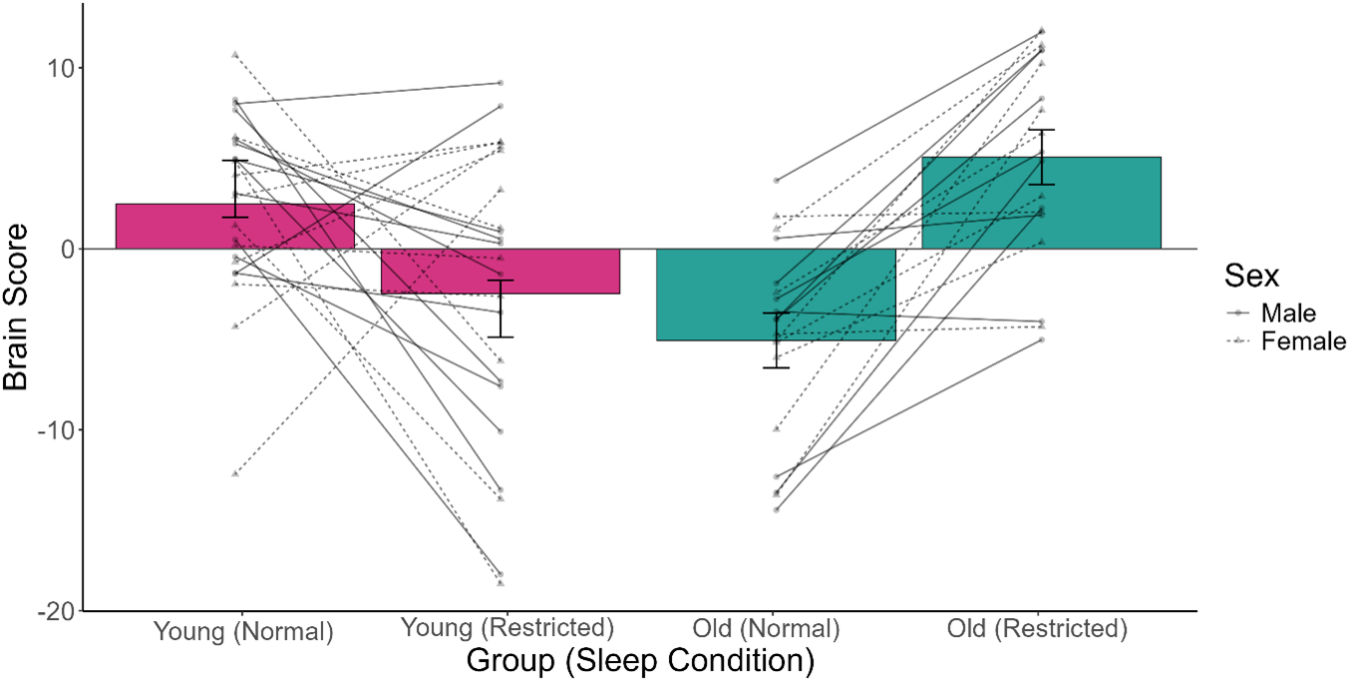
Mean brain scores from FC mean-centred PLS LV, for participants in the young and old age groups, in restricted and normal sleep conditions. Error bars represent 95% confidence intervals. Solid (male) and dashed (female) lines depict the change in the individual brain score values between sleep conditions.

**Fig. 2.**
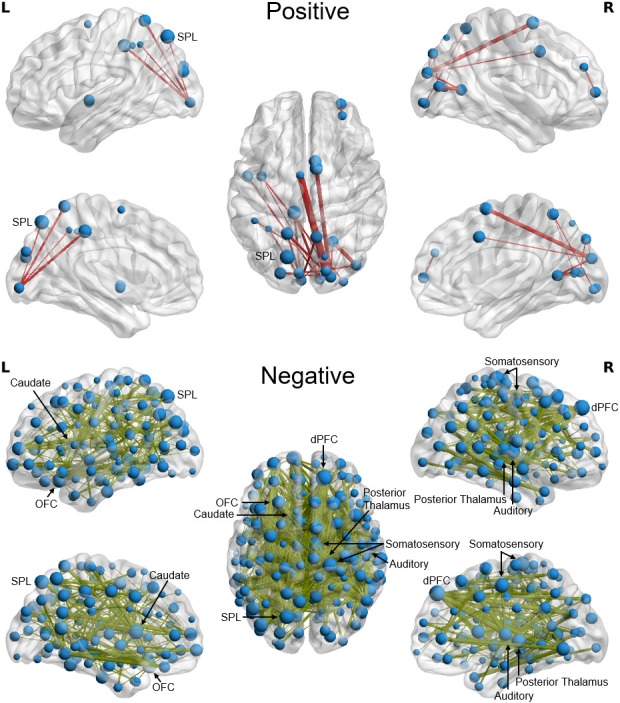
Reliable bootstrap ratios from FC mean-centred PLS LV with independent variables of age and sleep condition, depicted separately for positive and negative values. Positive values indicate connections that increase after acute sleep restriction for OA but decrease for YA, while negative values indicate connections that decrease after acute sleep restriction for OA but increase for YA. Connection size indicates the magnitude of the BSR. Highest degree regions are labelled (SPL: superior parietal lobule; OFC: orbitofrontal cortex; dPFC: dorsal prefrontal cortex).

**Fig. 3.**
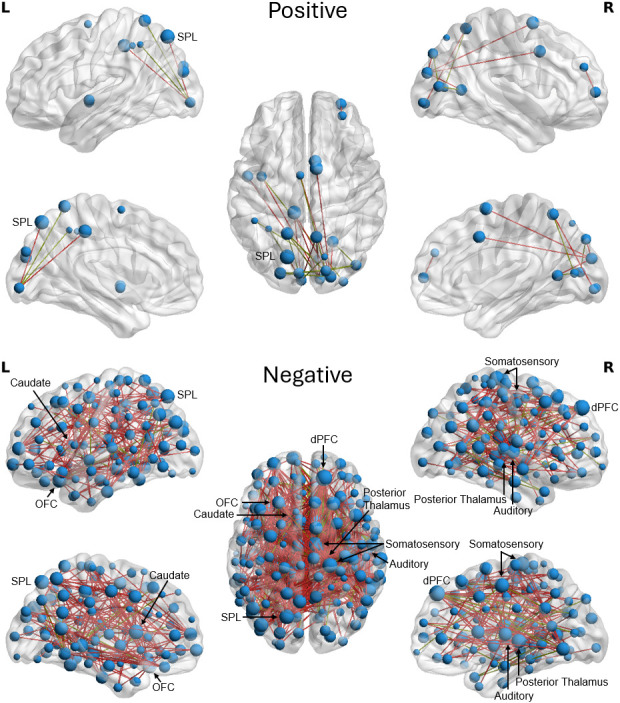
Mean connectivity difference between restricted and normal sleep conditions in YA for connections identified as reliable by the FC mean-centred PLS LV. Positive values indicate connections identified by the PLS that increase after acute sleep restriction for OA but decrease for YA, while negative values indicate connections that decrease after acute sleep restriction for OA but increase for YA. Highest degree regions are labelled (SPL: superior parietal lobule; OFC: orbitofrontal cortex; dPFC: dorsal prefrontal cortex).

A mean-centred PLS analysis was performed with FC as the dependent variable and independent variables of age group (YA vs. OA) and sleep condition (Restricted vs. Normal). This analysis identified one significant LV (*p* = .041) associated with a crossover interaction between age and sleep, whereby the effect of acute sleep restriction occurred in opposite directions for YA and OA (see Fig. 1). As seen by the lines showing the sleep effect for individuals by sex, for the YA males, all but two of these individuals followed the pattern identified by the LV, while YA females were more inconsistent. For OA, all but one of the males followed the pattern identified by the LV, and all of the females followed the pattern (see Fig. 1). The functional connections reliably associated with this LV were primarily negatively associated (682/710 connections; 96.056%), indicating that acute sleep restriction in OA was associated with reduced connectivity between these regions but increased connectivity in YA (see Fig. 2). A smaller number of connections were positively associated with the LV, indicating that these connections had increased connectivity during acute sleep restriction in OA but decreased connectivity during acute sleep restriction in YA. These connections were primarily made up of connections involving the occipital lobe. The most highly connected regions within this subnetwork of identified regions included the left hemisphere orbitofrontal cortex, superior parietal lobule, and caudate, and the right hemisphere dorsal prefrontal cortex, somatosensory cortex, auditory cortex, and posterior thalamus. Of the 710 identified connections, 21.972% were within the left hemisphere, 23.662% were within the right hemisphere, and 54.366% were interhemispheric. Furthermore, the mean proportion of within-network connections based on the Yeo-17 definition (Yeo et al., 2011) was .021, and the mean proportion of between-network connections was .030, representing no significant difference, *t*(16) = -.908, *p* = .377. Looking at the mean connectivity difference between restricted and normal sleep for these connections, OA exhibited a negative difference for all connections with negative BSRs and a positive difference for all connections with positive BSRs, consistent with the pattern identified by the LV. For YA, the majority (87.042%) of connections exhibited a positive difference for the negative BSRs and a negative difference for the positive BSRs, consistent with the pattern identified by the LV (see Fig. 3). A follow-up motion analysis identified a significant LV (*p* < .001) associated with motion, but the cosine similarity between the salience matrices of the motion model and the model of interest demonstrated that these models were not measuring the same effect (cosine similarity = -.188, permutation *p* = .438). In summary, the PLS analysis of FC identified a subnetwork that responds in one way to sleep restriction for OA, and in the opposite direction for YA.

**Fig. 4.**
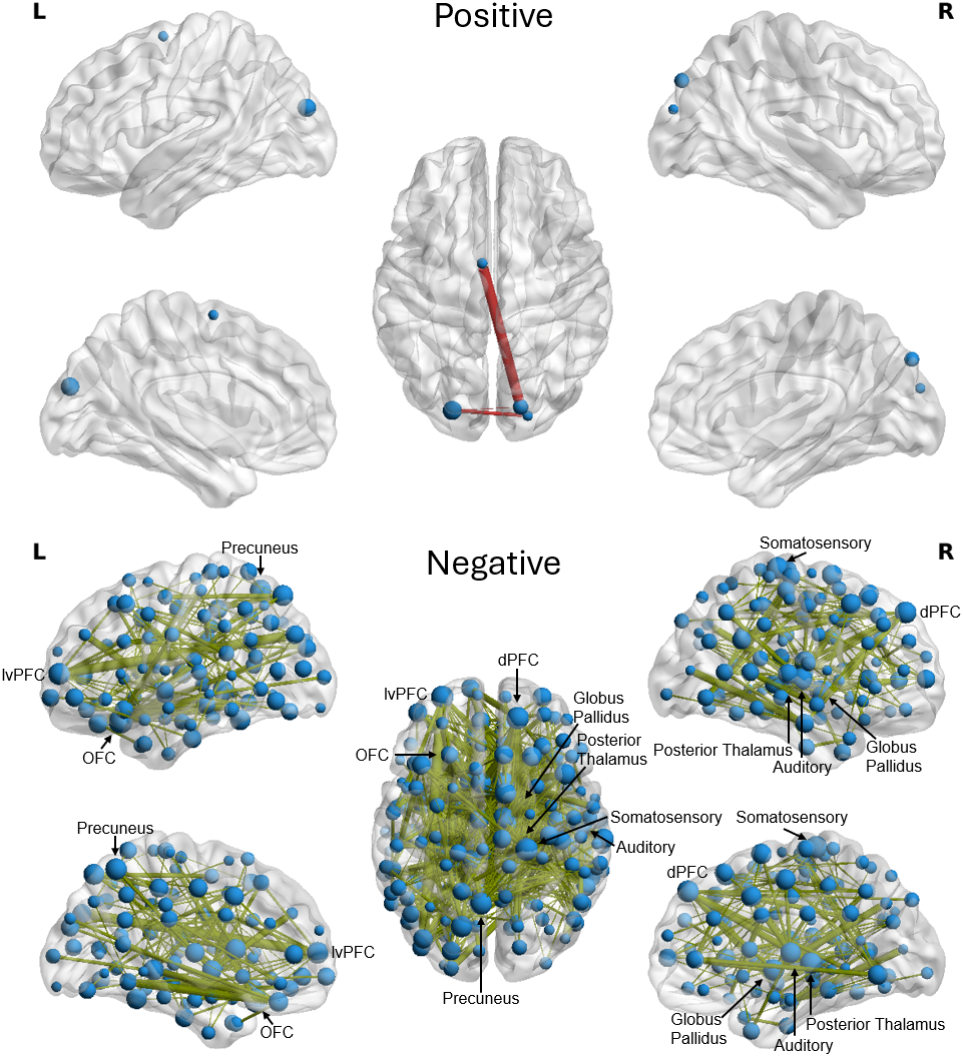
Functional connectivity cluster identified by the network-based statistic analysis, representing a significant difference between YA and OA on the sleep difference network (restricted minus normal sleep). Positive values indicate connections that increase after acute sleep restriction for OA but decrease for YA, while negative values indicate connections that decrease after acute sleep restriction for OA but increase for YA. Connection size indicates the magnitude of the t-score. Highest degree regions are labelled (lvPFC: lateral ventral prefrontal cortex; OFC: orbitofrontal cortex; dPFC: dorsal prefrontal cortex).

A similar analysis was conducted on the functional connectivity data using the network-based statistic (Zalesky et al., 2010). The functional connectivity sleep difference network was calculated by subtracting the normal sleep condition from the restricted sleep condition connectivity, and the network-based statistic was used to identify a subnetwork within this sleep difference network that differed between YA and OA groups (*p* = .040). The results of this analysis are shown in Figure 4, where positive values represent connections with a larger positive sleep effect for OA than for YA and negative values represent connections with a higher magnitude negative sleep effect for OA than for YA. This analysis identified a significant “difference of differences” (i.e., interaction) network conceptually overlapping with the network identified by the PLS. The most highly connected regions identified in this subnetwork included the left hemisphere orbitofrontal cortex and right hemisphere dorsal prefrontal cortex, somatosensory cortex, auditory cortex, and posterior thalamus, which were highlighted in the PLS analysis, as well as the left hemisphere lateral ventral prefrontal cortex and precuneus, and the right hemisphere globus pallidus. This subnetwork of 539 connections consisted of 20.965% LH connections, 22.820% RH connections, and 56.215% interhemispheric connections. Furthermore, the mean proportion of within-network connections based on the Yeo-17 definition (Yeo et al., 2011) was .027, and the mean proportion of between-network connections was .022, representing no significant difference, *t*(16) = .298, *p* = .769. Supplementary analyses with varied thresholds produced significant and similar results with *t* > 2.5 (Supplementary Fig. S1), but no significant network when *t* > 2.9. The convergence with the PLS finding suggests that any sensitivity to the threshold does not undermine the conclusions of the findings. In summary, the network-based statistic analysis of FC replicated the PLS findings, identifying an overlapping subnetwork that responds in one way to sleep restriction for OA, and in the opposite direction for YA.

### 3.2 Functional connectivity degree

**Fig. 5.**
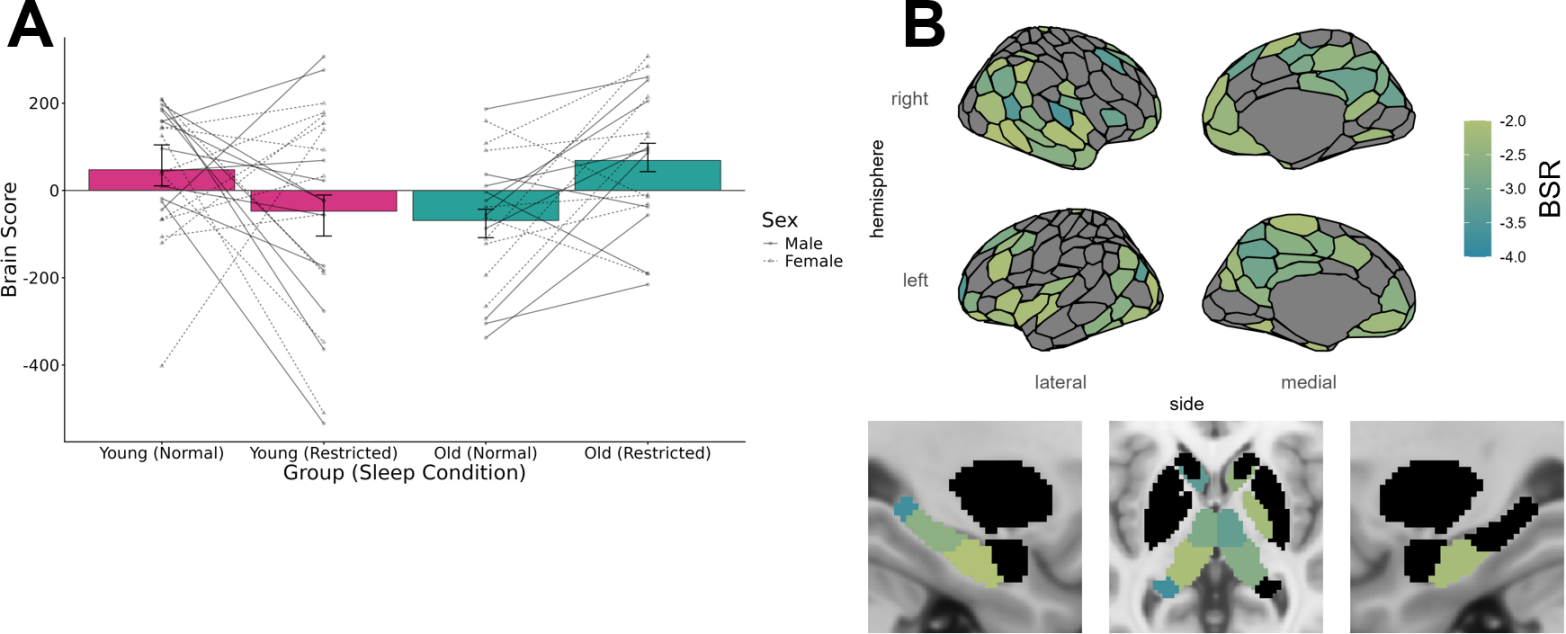
(A) Mean brain scores from FC degree mean-centred PLS LV, for participants in the young and old age groups, in restricted and normal sleep conditions. Error bars represent 95% confidence intervals. Solid (male) and dashed (female) lines depict the change in the individual brain score values between sleep conditions. (B) Reliable bootstrap ratios from FC degree mean-centred PLS LV with independent variables of age and sleep condition.

**Fig. 6.**
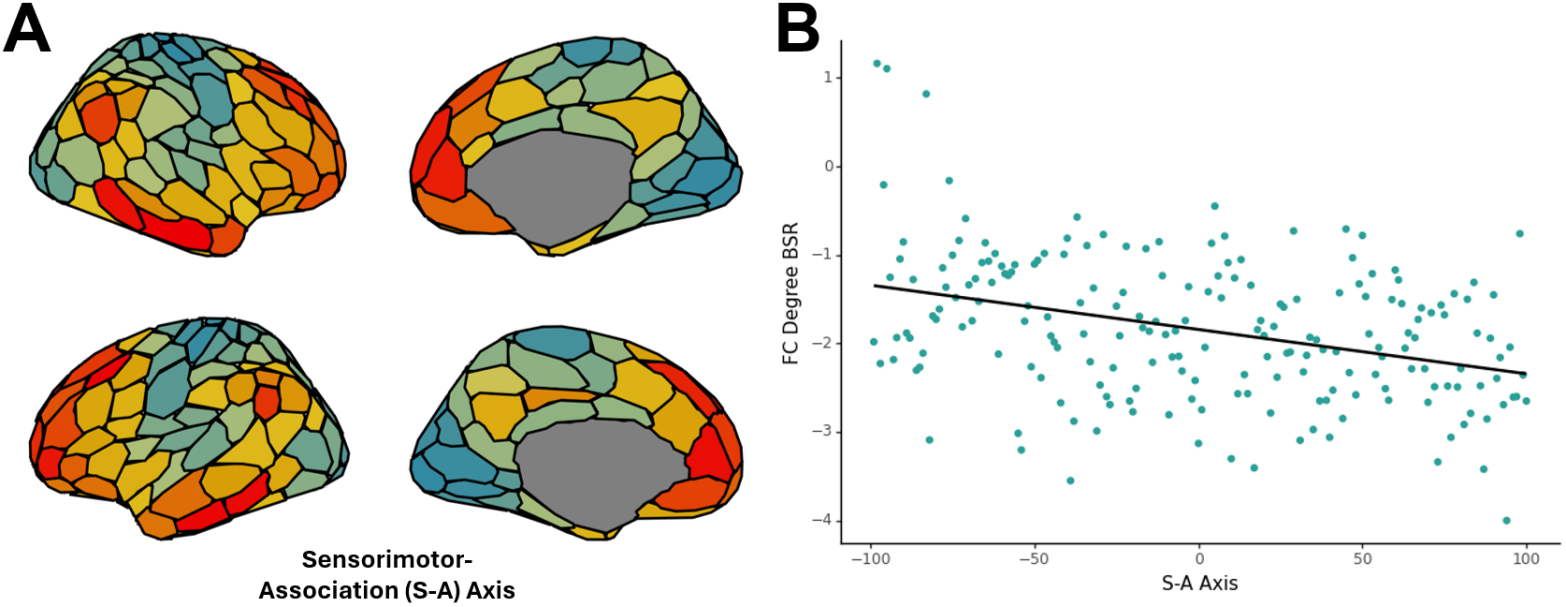
(A) For comparison with Figure 5B, the Sensorimotor-Association Axis (from blue/green sensorimotor regions to yellow/red association regions) from Luo et al. (2024). (B) Regions identified as reliable overlap with the association end of the S-A axis and BSRs are negatively correlated with the S-A Axis values, *R*(218) = -0.362, *p* < .001.

**Fig. 7.**
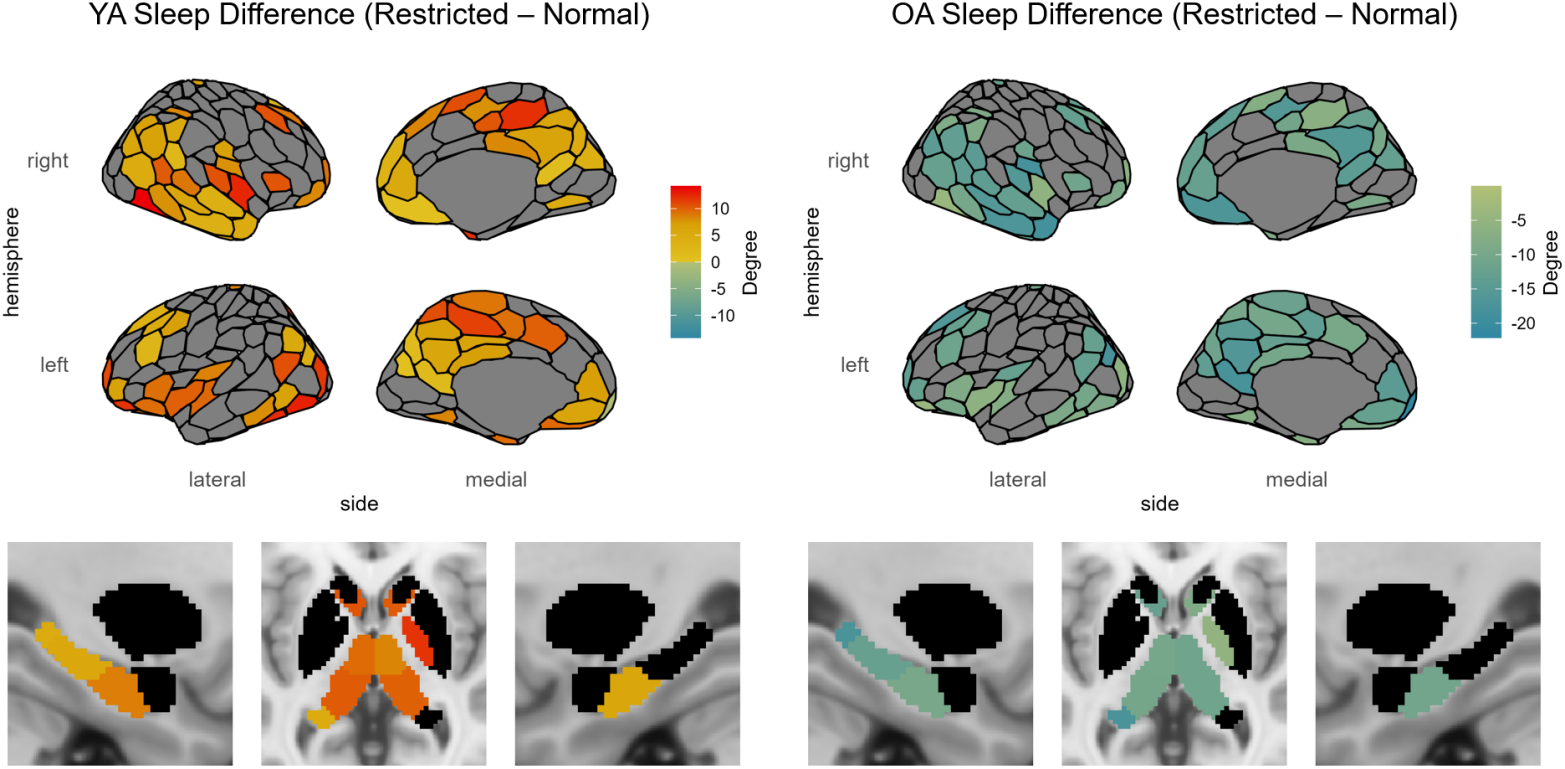
Mean FC degree difference between acutely restricted and normal sleep conditions in YA (left) and OA (right) for regions identified as reliable by the FC degree mean-centred PLS LV (see Fig. 5B). Whereas Figure 5B shows how reliable these regions are in the PLS analysis (BSRs), this figure shows the mean difference of the FC degree in these regions for each group.

A mean-centred PLS analysis was performed with FC degree (total regional connectivity weights) as the dependent variable and independent variables of age group and sleep condition. This analysis identified one significant LV (*p* = .021) associated with a crossover interaction between age and sleep, whereby the effect of acute sleep restriction occurred in opposite directions for YA and OA (see Fig. 5A). As seen by the lines showing the sleep effect for individuals by sex, for the YA males, all but three of these individuals followed the pattern identified by the LV, while YA females were much more inconsistent. For OA, all but two of the males followed the pattern identified by the LV, and all but two of the females followed the pattern (see Fig. 5A). The functional connections reliably associated with this LV were all negatively associated, indicating that acute sleep restriction in OA was associated with reduced degree in these regions, but increased degree in YA (see Fig. 5B). The pattern of cortical regions identified by this LV resembles the association end of the Sensorimotor-Association (S-A) Axis (e.g., Luo et al., 2024; see Fig. 6A), and indeed, the cortical BSR values from this LV were negatively correlated with the S-A Axis values, *R*(218) = -0.362, *p* < .001 (see Fig. 6B). Looking at the mean degree difference between restricted and normal sleep for these regions, OA exhibited a negative difference for all regions and YA exhibited a positive difference for these regions, consistent with the pattern identified by the LV (see Fig. 7). A follow-up motion analysis identified no significant LV (*p* = .094) associated with motion. In summary, the PLS analysis of FC degree identified regions whose connectivity responds in one way to sleep restriction for OA, and in the opposite direction for YA.

### 3.3 Dynamic functional connectivity

**Fig. 8.**
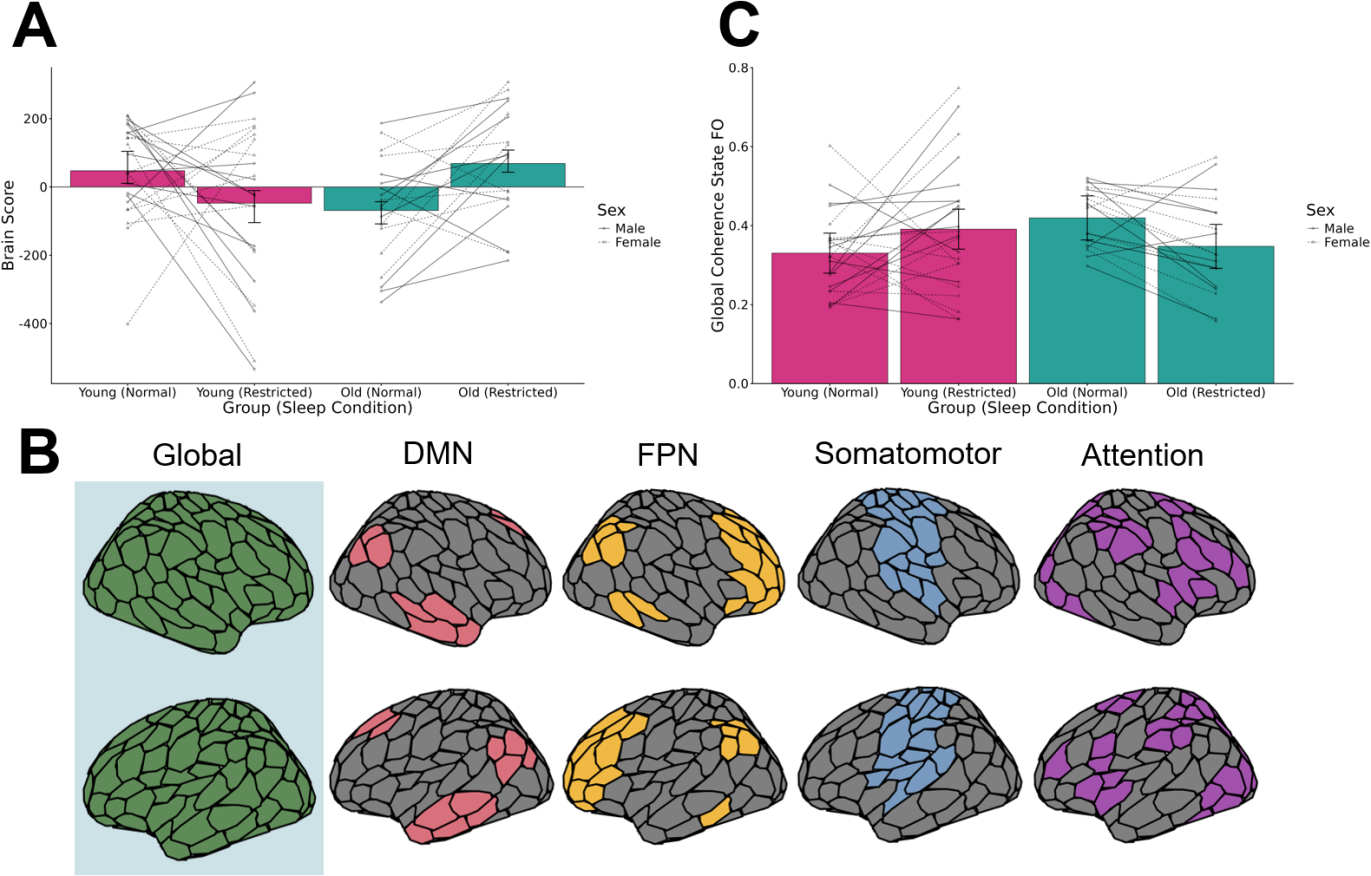
(A) Mean brain scores from LEiDA fractional occupancy mean-centred PLS LV, for participants in the young and old age groups, in restricted and normal sleep conditions. Error bars represent 95% confidence intervals. Solid (male) and dashed (female) lines depict the change in the individual brain score values between sleep conditions. (B) Reliable bootstrap ratios from LEiDA fractional occupancy mean-centred PLS LV with independent variables of age and sleep condition (blue highlighting indicates a significant negative BSR for the Global Coherence State). (C) Mean fractional occupancy of the Global Coherence State in each of the conditions, with 95% confidence intervals from a linear mixed effects model producing a significant interaction between sleep (normal) and age (young), *t* = -2.701, *d* = 1.088 (Cohen’s *d*-like effect size parameter calculated as described by Westfall et al., 2014).

The LEiDA dynamic FC analysis identified five states, interpreted as Global Coherence, Default Mode Network (DMN), Frontoparietal Network, Somatomotor, and Attention states. The fractional occupancy of these states (relative time spent in each state) was analyzed using a mean-centred PLS with fractional occupancy as the dependent variable and independent variables of age group and sleep condition. One significant LV (*p* = .018) was identified, exhibiting the same cross-over interaction association as shown previously (see Fig. 8A). This LV identified a decrease in the amount of time spent in the Global Coherence state for OA after restricted sleep, and an opposite effect of acute sleep restriction on YA (see Fig. 8B for BSRs and Fig. 8C for mean fractional occupancy of the Global Coherence state). In the YA group, 5 of 12 males and 6 of 11 females followed the pattern of association identified by the LV, and in the OA group, 8 of 10 males and 8 of 9 females followed the pattern of association identified by the LV (see Fig. 8A). A follow-up motion analysis identified a significant LV (*p* < .001) associated with motion, but the cosine similarity between the salience matrices of the motion model and the model of interest demonstrated that these models were not measuring the same effect (cosine similarity = -.726, permutation *p* = .204).

The dwell time of the dynamic FC states was also analyzed using a mean-centred PLS with dwell time as the dependent variable and independent variables of age group and sleep condition. One significant LV (*p* = .042) was identified that produced the same pattern of results as for fractional occupancy, exhibiting the same cross-over interaction associated with a decrease in the amount of time spent in the Global Coherence state for OA after acutely restricted sleep, and an increase in the amount of time spent in the Global Coherence state for YA after acutely restricted sleep. A follow-up motion analysis identified a significant LV (*p* < .001) associated with motion, but the cosine similarity between the salience matrices of the motion model and the model of interest demonstrated that these models were not measuring the same effect (cosine similarity = -.727, permutation *p* = .138).

An analysis of the transition probability matrix, representing the likelihood of each possible state transition, was performed using a mean-centred PLS with independent variables of age and sleep, but produced no significant LV. In summary, the dynamic FC analyses demonstrated that older adults spend less time in the global coherence state after acutely restricted sleep compared with normal sleep, while younger adults exhibit the opposite pattern, spending more time in the global coherence state after acutely restricted sleep compared with normal sleep.

### 3.4 Network modularity

**Fig. 9.**
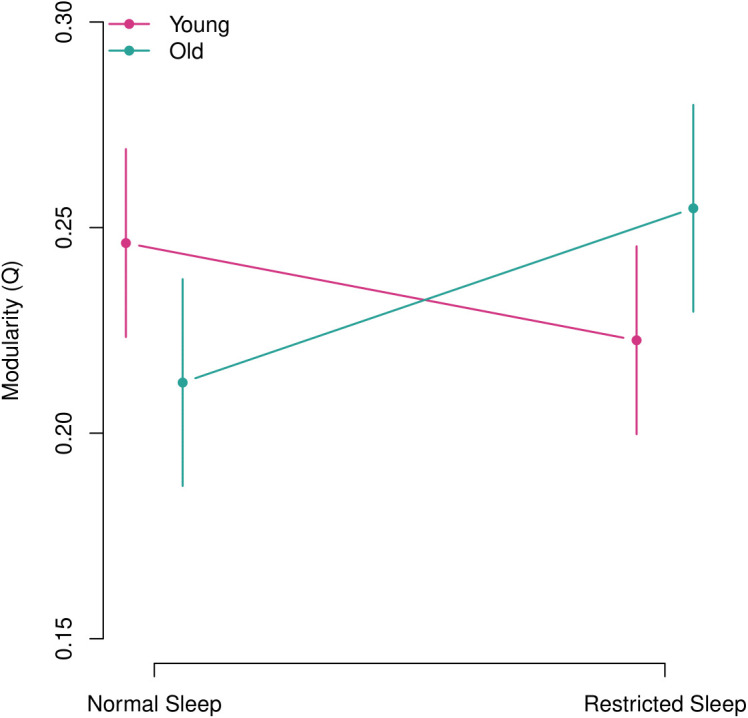
FC modularity as a function of age and sleep. Error bars represent the 95% confidence interval.

A network modularity analysis was conducted based on functional connectivity modules identified by the Louvain algorithm. High modularity indicates that the network has strong connectivity within modules but weaker connectivity between modules. A linear mixed effects (Bates et al., 2015) model identified a significant crossover interaction between age and sleep, *t* = 3.067, *d* = .420 (Cohen’s *d*-like effect size parameter calculated as described by Westfall et al., 2014), whereby modularity increased in the restricted sleep condition for OA but decreased for YA (see Fig. 9 and Table 1). This model included subject as a random effect (including sleep as a random effect was also tested but did not converge). Additional models varying the resolution parameter used to calculate modularity were also inspected, which produced the same significant crossover interaction between age and sleep (see Supplementary Figs. S2–S3 and Tables S1–S2). Although age was not significant in the LMM, the 95% confidence intervals indicate that OA had less modularity than YA in the normal sleep condition, and that under restricted sleep conditions, YA modularity was reduced to normal OA levels. Additionally, we demonstrated that OA modularity is increased to normal YA levels in the restricted sleep condition, representing a novel discovery. An additional analysis was performed with motion added to the existing model, which produced a significant effect of motion but the other effects produced the same pattern as the original model. In summary, the modularity analysis demonstrated that older adults exhibit greater modularity after acute sleep restriction compared with normal sleep, while younger adults show the opposite pattern, exhibiting less modularity after acute sleep restriction compared with normal sleep.

**Table 1.** Linear Mixed Model results with modularity as the dependent variable and independent variables age and sleep plus their interaction, including a random intercept for participants.

	Errors		
Random Effects	Variance		SD
**Participants**			
Intercept	6.291 × 10^-4^		.025
**Residual**	2.411 × 10^-3^		.049
Fixed Effects	Estimate (OR)	Std. Error	t-value
Intercept	.246	.012	21.417
Sleep (Restricted)	-.024	.014	-1.633
Age (Old)	-.034	.017	-1.985
Sleep (Restricted) × Age (Old)	.066	.022	*3.067

Note. An asterisk denotes a significant effect.

### 3.5 Voxel-wise functional connectivity

**Fig. 10.**
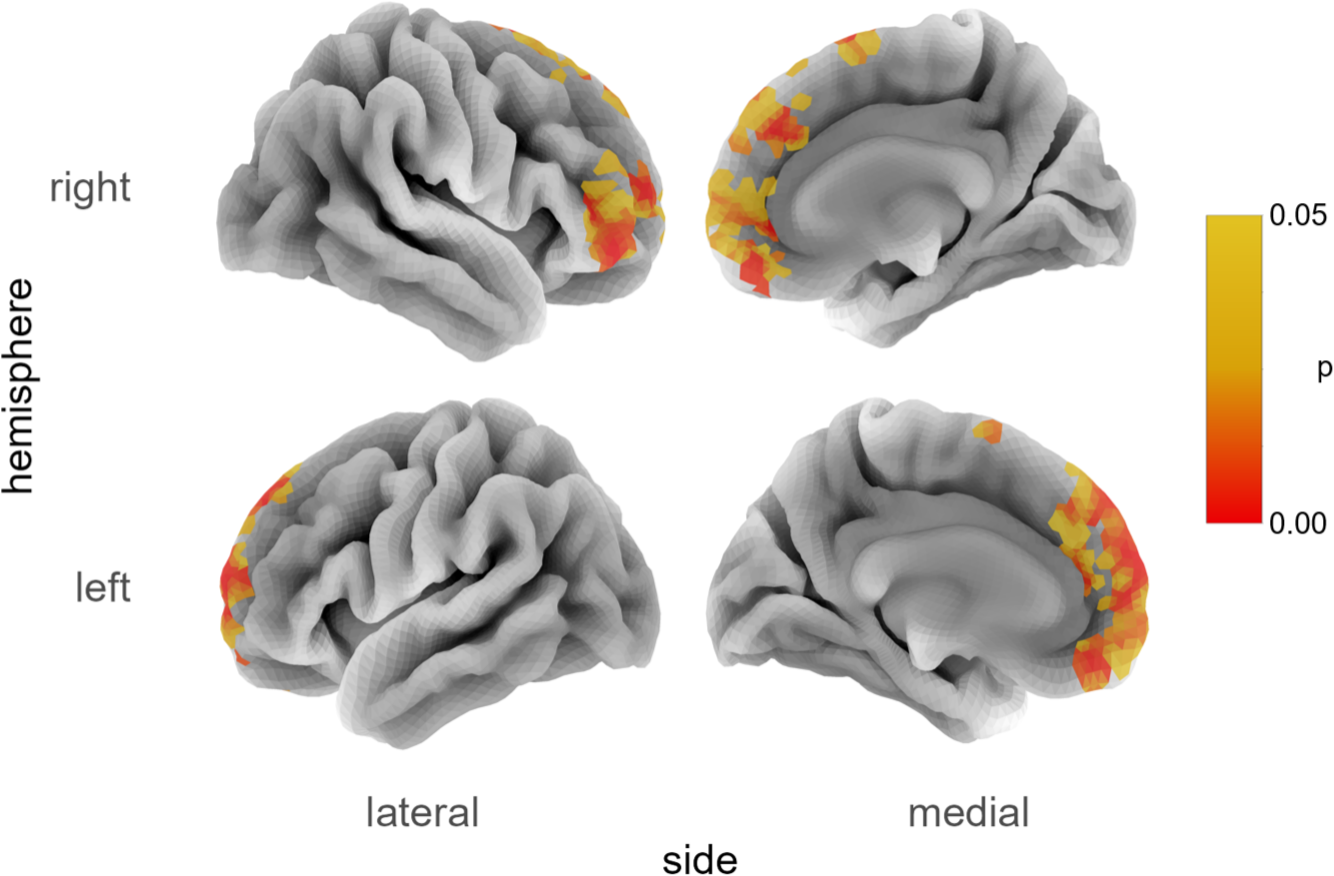
Voxels exhibiting a sleep condition-dependent difference in their FC connectivity profile as identified by multivariate distance matrix regression for YA.

Multivariate distance matrix regression analyses were performed on the voxel-wise functional connectivity matrices comparing sleep conditions within age groups, and on the voxel-wise functional connectivity sleep difference matrices (subtracting the connectivity values for normal sleep from those obtained in the restricted sleep condition) between age groups. Of these analyses, only the sleep condition comparison within YA identified significant voxels, identifying a cluster of voxels primarily localized within the bilateral medial, orbitofrontal, and dorsolateral PFC (see Fig. 10). Additional analyses with increased cluster permutations (750 and 1000) were performed, which again produced significant results in the same pattern as shown in Figure 10 (see Supplementary Figs. 4 and 5). In summary, young adults exhibited a differential response to acute sleep restriction in the functional connectivity of voxels primarily in the prefrontal cortex.

## 4 Discussion

We have identified multiple aspects of the functional connectivity network that exhibit opposite effects of acute sleep restriction when comparing young and older adults. This effect was observed in the functional connectivity values, functional connectivity degree (total connection values to brain regions), dynamic functional connectivity fractional occupancy and dwell time (how much time an individual spent in each distinct brain state), and whole brain modularity (the extent to which segregated modules are more connected within themselves than between themselves). Both the mean-centred PLS and the network-based statistic methods identified a significant FC subnetwork associated with acute sleep restriction, composed primarily of connections that were weaker after acute sleep restriction for older adults but, conversely, were stronger after acute sleep restriction for younger adults. The most highly implicated regions within this subnetwork included left hemisphere orbitofrontal cortex and right hemisphere dorsal prefrontal cortex, somatosensory cortex, auditory cortex, and posterior thalamus, which both PLS and the network-based statistic identified. Hippocampal–neocortical connections were implicated, with the largest number of these represented by hippocampal connections to the prefrontal cortex and orbitofrontal cortex connections, as well as a smaller number of connections between the hippocampus and the insula, temporal pole, somatosensory cortex, and secondary somatosensory cortex. Using graph theory to investigate the regional network effects of acute sleep restriction, we found a large number of regions where functional connectivity degree decreased after acute sleep restriction for older adults but increased after acute sleep restriction for young adults. The regions identified were primarily composed of association regions. A dynamic functional connectivity analysis identified five states, their fractional occupancies, and their transition probabilities. Older adults spent less time (measured via fractional occupancy and dwell time) in the global coherence state after acute sleep restriction, while young adults spent more time in this state after acute sleep restriction. Furthermore, functional connectivity modularity also demonstrated a crossover interaction with age and sleep, whereby young adults exhibited reduced modularity after acute sleep restriction. For young adults, modularity fell to the level of older adults under normal sleep conditions, while older adults exhibited enhanced modularity after acute sleep restriction, boosted to levels corresponding to young adults under normal sleep conditions. While we were not able to identify age differences in voxel-wise functional connectivity profiles using multivariate distance matrix regression, we identified a cluster of significant voxels exhibiting an acute sleep difference for young adults, localized to the bilateral medial prefrontal cortex, orbitofrontal cortex, and dorsolateral prefrontal cortex. This granular level of detail about the network effect of acute sleep restriction in YA has not been reported in the literature and represents an important contribution to network neuroscience sleep research.

These findings represent a novel demonstration of a consistent cross-over interaction between the effects of age and acute sleep loss on brain network measures. Finding an interaction of these variables has been attempted previously without success (Nilsonne et al., 2017), but we were able to observe the interaction across numerous metrics by focusing on edge- and region-centric analyses, which allow for the network to be inspected in finer detail. Our dynamic functional connectivity analyses also represent a novel investigation in this domain, which produced a consistent interaction when looking at state fractional occupancy and dwell time, whereby older adults spent less time in the Global Coherence state after sleep restriction. Our previous research has identified less time in the Global Coherence state as a marker of preserved visual working memory in older adults, highlighting this measure as a positive brain feature in ageing (Neudorf et al., 2025). We also discovered that the whole-brain metric of modularity exhibited this cross-over interaction. We demonstrated that OA had less modularity than YA in the normal sleep condition, replicating past research (Song et al., 2014), and under restricted sleep conditions YA modularity was reduced to normal OA levels, replicating others (Zhou et al., 2017). Our observed decrease in YA modularity under acutely restricted sleep has also been demonstrated in other research (Ben Simon et al., 2017). Additionally, we demonstrated that OA modularity in the acutely restricted sleep condition is increased to normal YA levels, representing a novel discovery that was not possible in past research that did not include all four conditions. Given that modularity is associated with better cognitive performance (Bertolero et al., 2018), these findings suggest that acute sleep restriction may induce compensatory reorganization of functional connectivity for older adults. The dataset does not include cognitive measures for both sleep conditions, but this possibility should be investigated in future research. Compensatory reactions of the brain to acute sleep restriction should not be surprising, given the past research on young adults that has demonstrated brain function compensation in response to sleep loss (Caldwell et al., 2004; Chengyang et al., 2017; Drummond et al., 2000; Tian et al., 2024).

The idea that *acute* loss of sleep may result in temporary positive effects may seem counterintuitive, given what we know about the negative effects of *chronic* loss of sleep. However, the chronic effects of sleep loss in older age are cumulative across the lifespan, and have the greatest impact in younger adulthood and middle age, increasing the risk of dementia and cognitive decline, whereas chronic sleep loss in older adults does not impact risk of cognitive decline or dementia consistently or to the same extent as earlier in the lifespan (as reviewed by Scullin & Bliwise, 2015). Furthermore, there are positive features of acute sleep loss that have been observed in previous research. For example, although chronic lack of sleep is associated with increased depression symptoms (Fang et al., 2019; Krakow et al., 2000), acute sleep deprivation can temporarily reduce symptoms of depression in around half of depression and posttraumatic stress disorder patients (Thase et al., 2017).

It may also be surprising, at first, that older adult brain networks consistently respond to acute sleep restriction in an opposite way to young adults. However, research has demonstrated that cognitive abilities are negatively affected by acute sleep loss for young adults, while older adults are relatively or completely spared from these effects (Duffy et al., 2009; Gerhardsson et al., 2019; Scullin & Bliwise, 2015). Similarly, the benefits of napping on cognitive function are often only seen for young and middle-aged adults, and the benefits of sleep for memory consolidation are stronger for younger adults than for older adults (Scullin & Bliwise, 2015). Additionally, recent research from our laboratory has shown multiple ways in which the healthy ageing brain network reorganizes to support cognitive ability, utilizing a distinct network regime that is different in fundamental ways from the healthy young adult brain (Heisz et al., 2015; Neudorf et al., 2024, 2025). For these reasons, it could be expected that the way the young and older adult brain networks adapt to restricted sleep are also fundamentally different in the ways we have identified here.

Our results provide insight into theories that have been developed to explain the discrepancy between young and older adults in the effects of acute sleep restriction on cognitive abilities. As we have highlighted, older adults are relatively or completely spared from the effects of acute sleep restriction, unlike young adults (Scullin & Bliwise, 2015). Our findings support the compensation theory most strongly, as we did not simply observe in older adults the absence or decrease of an acute sleep restriction effect seen for young adults, as would be expected based on the reduced sleep need and functional weakening theories. Rather, we consistently identified positive brain network features that increased after acute sleep restriction for older adults and decreased for younger adults. This finding is consistent with the theory that the brain compensates for changes due to age by reorganizing the brain in a fundamental way.

Rather than these differences being localized to specific brain regions, these findings appear to point to a broader network effect of decreased functional connectivity for OA under acutely restricted sleep conditions, affecting association areas more than sensorimotor areas as demonstrated in Figures 5 and 6. In addition, the amount of time spent in the Global Coherence state decreased under acutely restricted sleep for OA. Although no remaining state (DMN, FPN, Somatomotor, and Attention) was singled out as being more highly represented under acutely restricted sleep for OA, the decreased time in the Global Coherence state means that more time was spent in the other more organized states in a shared fashion. Taken together, the decreased association connections and increased time spent in organized functional connectivity dynamics appear to have produced a more efficiently organized modular network organization following acute sleep restriction for OA as demonstrated by the modularity analysis. Future neuroscience research with the ability to test the mechanisms of this complex interaction between sleep, brain dynamics, and network organization would be valuable for gaining a more mechanistic understanding of these novel results.

These results make it clear that the ageing brain responds very differently to the amount of sleep it gets than younger adults. From a complex systems perspective, the trajectories of young adult and older adult brains travel in opposite directions along modularity and organized dynamics dimensions in response to acute sleep loss. That is to say, whereas young adult brains become less modular with less organized dynamic FC under acutely restricted sleep, older adult brains instead become more modular and spend more time in organized dynamic functional states. If the sleep need theory or functional weakening theory were applicable to this domain, we would expect a negative trajectory for young adults with a lesser or undetectable change for older adults, but instead we observe this opposite trajectory towards modularity and dynamics mirroring what we would expect at a young adult healthy baseline. This effect fits with the definition of a compensatory response as we observed an increase in modularity, which has been identified as a positive feature in adult brains (Bertolero et al., 2018), supporting more strongly the compensatory theory of sleep loss resilience with age.

### 4.1 Limitations and future directions

The dataset used for this analysis did not include sufficient cognitive assessment measures, and did not include measurements in both sleep conditions to assess the impact of acute sleep restriction on cognitive performance. For this reason, the analyses we conducted here should be replicated using data that include cognitive performance measures pre- and post-acutely restricted sleep, in order to determine which of the brain network responses to acute sleep restriction may be compensatory. These could include measures related to memory in order to investigate the impact of the hippocampal–neocortical connection alterations. Furthermore, a larger sample size with more variation in age (e.g., filling in the gap between 30 and 65 years old that is missing from the current research) would help to increase the power of these analyses and allow for detection of more subtle effects (e.g., dynamic FC transition probability, the multivariate distance matrix regression effect of the undetected acute sleep restriction effect in OA, and the voxel-wise interaction between sleep and age effects using this method). In particular, looking at middle age and the transition to older adulthood could uncover how the brain’s response to acute sleep restriction transitions over time into such opposite patterns of acute sleep restriction-induced reorganization. As indicated by the authors who curated this dataset, participants were excluded who indicated that they experienced insomnia symptoms or excessive snoring, rather than excluding based on a definitive sleep disorder diagnosis. This allows the findings we report to be conservatively protected from confounds related to these symptoms, but also means that the increased prevalence of these symptoms in the ageing population (Fernandes et al., 2024) is not captured by this research. Future research could investigate whether the presence of these symptoms alters the effects we report here. The modularity analysis produced a significant effect of head motion. However, the inclusion of this variable in the model with age and sleep did not change the effects of these variables of interest. This indicates that modularity was influenced by motion, but not in a way that undermined the findings discussed here. Finally, including an analysis of biomarkers for dementia (e.g., amyloid, tau, alpha-synuclein, and neurodegeneration) would be beneficial, particularly because sleep disturbances are risk factors and symptoms of several distinct neurodegenerative diseases. For this reason, these biomarkers could confound the pure effects of age and should be included in future research.

## 5 Conclusions

We have demonstrated yet another way in which the older adult brain represents a distinct brain network regime from the young adult brain. Young and older adults’ brain networks respond to acute sleep loss conditions in opposite ways, as measured using functional connectivity, degree, modularity, and dynamic functional connectivity. The opposing effects were observed in multiple brain network variables, suggesting a consistent and fundamental difference in the effects of acute sleep restriction on young and older adults. Further research should continue to investigate these effects and how they relate to preserved cognitive ability and brain health.

## Supplementary Material

Supplementary Material

## Data Availability

Data were downloaded from OpenfMRI (https://openfmri.org/dataset/ds000201/). Code used to produce analyses are available via github repository (https://github.com/McIntosh-Lab/SleepyBrain_analyses).
