# Supplementary material for: Young and old adult brains experience opposite effects of acute sleep restriction on the functional connectivity network
Source: Imaging Neurosci (Camb). 2026 Jun 18;4:IMAG.a.1278. doi: 10.1162/IMAG.a.1278 (PMC13281775; doi:10.1162/IMAG.a.1278)
Supplement: Supplementary Material [file IMAG.a.1278_supp.pdf]

Supplementary Materials

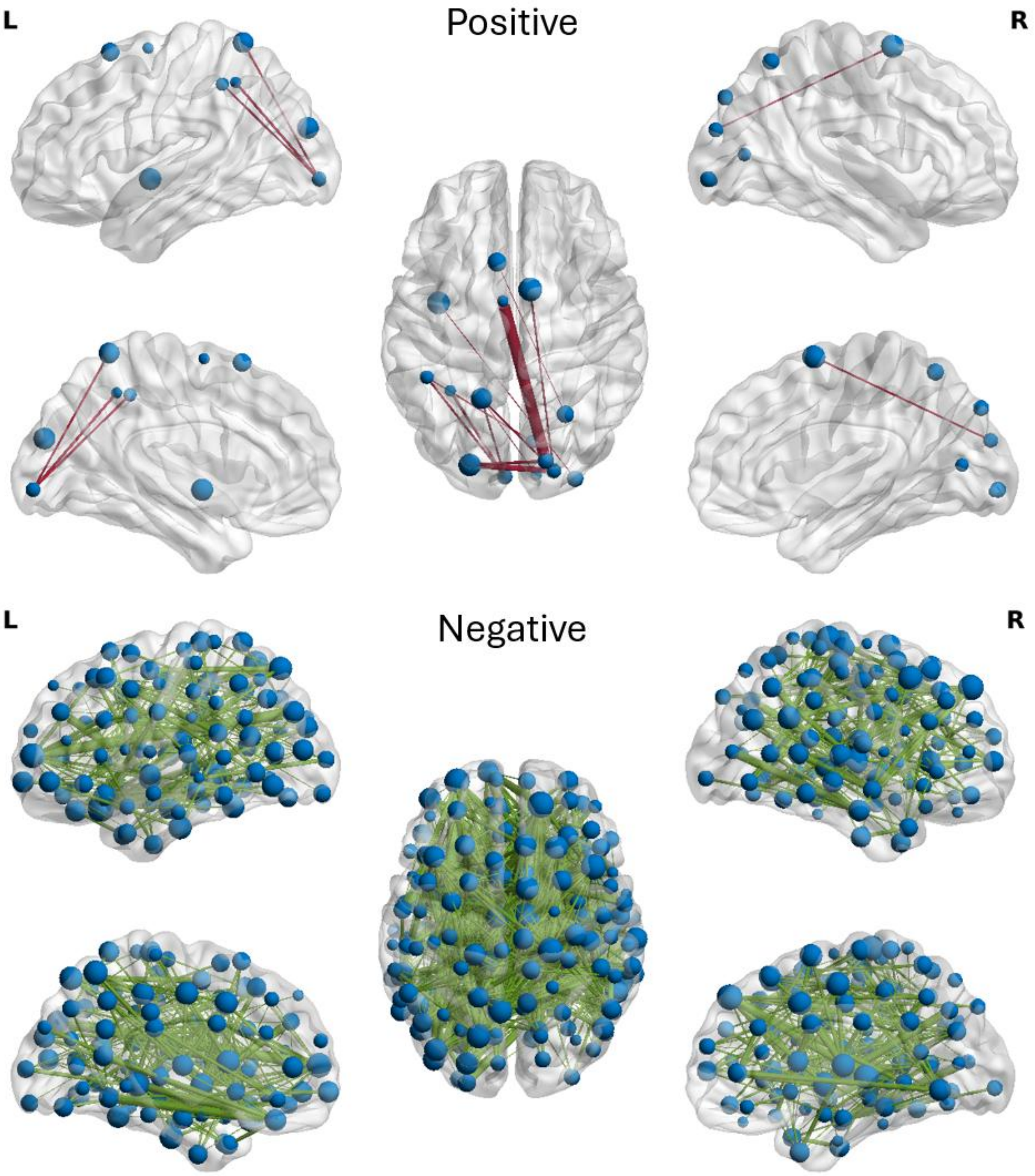

Supplementary Figure 1. Functional connectivity cluster identified by the network-based statistic analysis ($t > 2.5$), representing a significant difference between YA and OA on the sleep difference network (restricted minus normal sleep). Positive values indicate connections that increase after acute sleep restriction for OA but decrease for YA, while negative values indicate connections that decrease after acute sleep restriction for OA but increase for YA. Connection size indicates the magnitude of the t-score.

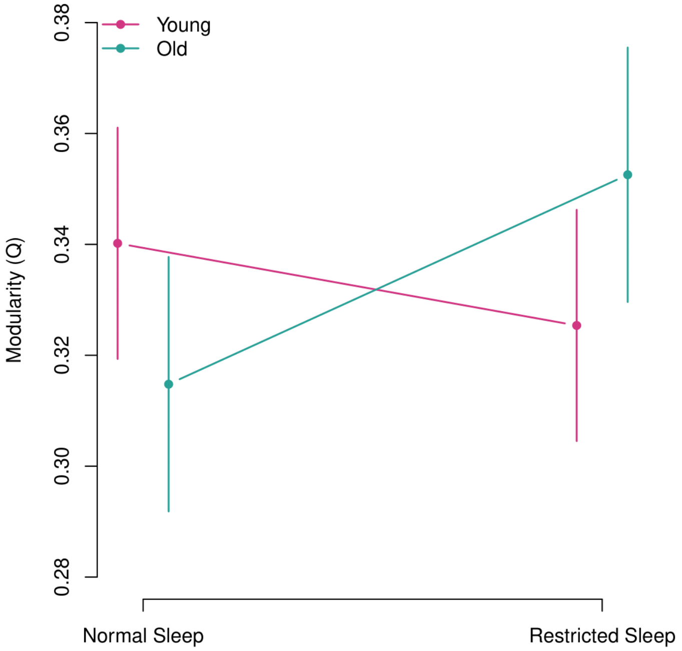

Supplementary Figure 2. FC modularity as a function of age and sleep. FC modularity calculated with resolution parameter set to 0.75. Error bars represent the 95% confidence interval.

Supplementary Table 1. Linear Mixed Model results with modularity (resolution parameter set to 0.75) as the dependent variable and independent variables age and sleep plus their interaction, including a random intercept for participants.

	Errors		
Random Effects	Variance		SD
**Participants**			
Intercept	7.254×10^{-4}		.027
**Residual**	1.804×10^{-3}		.042
Fixed Effects	Estimate (OR)	Std. Error	t-value
Intercept	.340	.010	32.441
Sleep (Restricted)	-.015	.013	-1.183
Age (Old)	-.025	.016	-1.630
Sleep (Restricted) × Age (Old)	.053	.019	*2.824

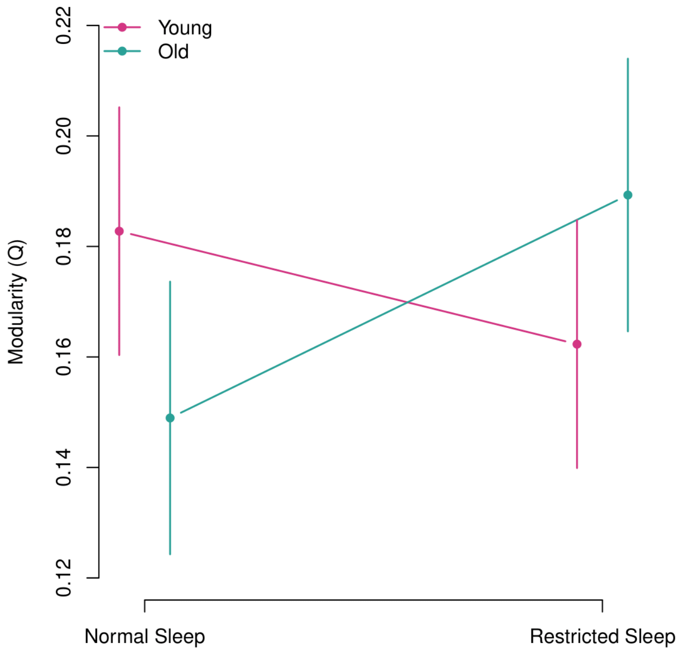

Supplementary Figure 3. FC modularity as a function of age and sleep. FC modularity calculated with resolution parameter set to 1.25. Error bars represent the 95% confidence interval.

Supplementary Table 2. Linear Mixed Model results with modularity (resolution parameter set to 1.25) as the dependent variable and independent variables age and sleep plus their interaction, including a random intercept for participants.

	Errors		
Random Effects	Variance		SD
**Participants**			
Intercept	4.302×10^{-4}		.021
**Residual**	2.494×10^{-3}		.050
Fixed Effects	Estimate (OR)	Std. Error	t-value
Intercept	.183	.011	16.209
Sleep (Restricted)	-.020	.015	-1.389
Age (Old)	-.034	.017	*-2.017
Sleep (Restricted) × Age (Old)	.061	.022	*2.777

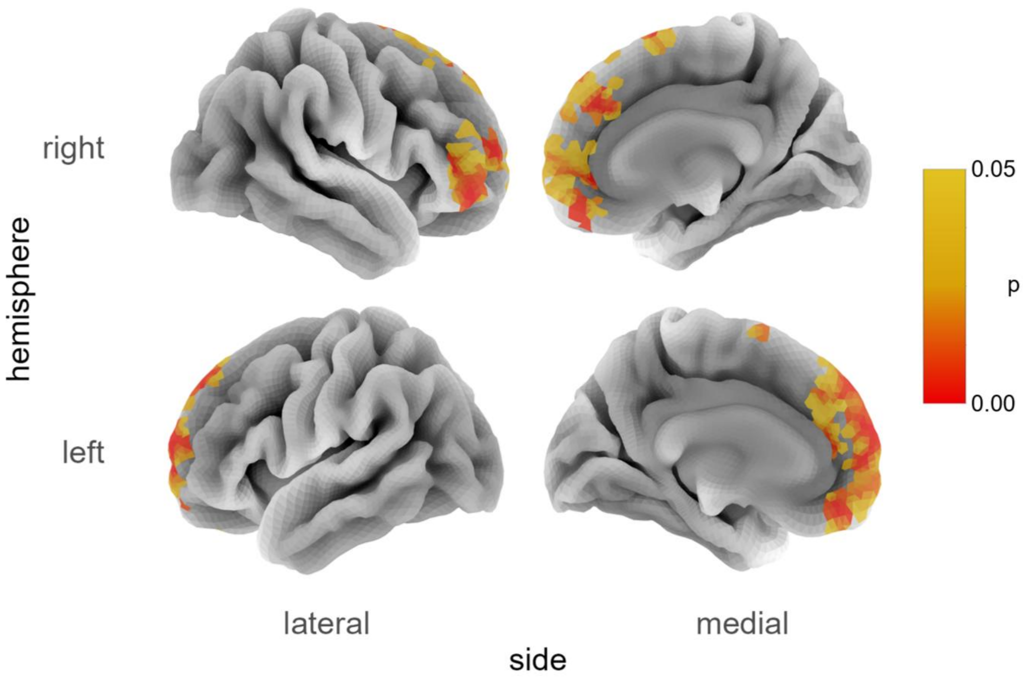

Supplementary Figure 4. Voxels exhibiting a sleep condition dependent difference in their FC connectivity profile as identified by multivariate distance matrix regression for YA, using 750 cluster permutations.

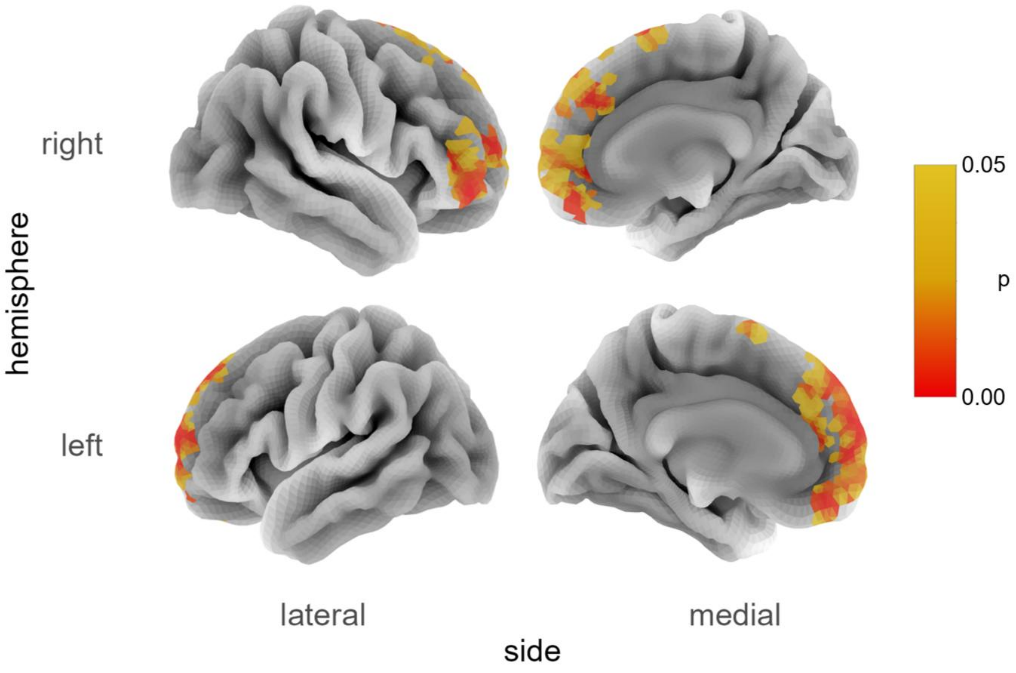

Supplementary Figure 5. Voxels exhibiting a sleep condition dependent difference in their FC connectivity profile as identified by multivariate distance matrix regression for YA, using 1000 cluster permutations.
